# Soy Formula and Epigenetic Modifications: Analysis of Vaginal Epithelial Cells from Infant Girls in the IFED Study

**DOI:** 10.1289/EHP428

**Published:** 2016-08-19

**Authors:** Sophia Harlid, Margaret Adgent, Wendy N. Jefferson, Vijayalakshmi Panduri, David M. Umbach, Zongli Xu, Virginia A. Stallings, Carmen J. Williams, Walter J. Rogan, Jack A. Taylor

**Affiliations:** 1 Epigenetics and Stem Cell Biology Laboratory,; 2 Epidemiology Branch,; 3 Reproductive and Developmental Biology Laboratory, and; 4 Biostatistics and Computational Biology Branch, National Institute of Environmental Health Sciences, National Institutes of Health, Department of Health and Human Services, Research Triangle Park, North Carolina, USA; 5 Division of Gastroenterology, Hepatology and Nutrition, Children’s Hospital of Philadelphia, University of Pennsylvania Perelman School of Medicine, Philadelphia, Pennsylvania, USA

## Abstract

**Background:**

Early-life exposure to estrogenic compounds affects the development of the reproductive system in rodent models and humans. Soy products, which contain phytoestrogens such as genistein, are one source of exposure in infants fed soy formula, and they result in high serum concentrations.

**Objectives:**

Our goal was to determine whether soy exposure is associated with differential DNA methylation in vaginal cells from soy-fed infant girls.

**Methods:**

Using the Illumina HumanMethylation450 BeadChip, we evaluated epigenome-wide DNA methylation in vaginal cells from four soy formula–fed and six cow formula–fed girls from the Infant Feeding and Early Development (IFED) study. Using pyrosequencing we followed up the two most differentially methylated sites in 214 vaginal cell samples serially collected between birth and 9 months of age from 50 girls (28 soy formula–fed and 22 cow formula–fed). With a mouse model, we examined the effect of neonatal exposure to genistein on gene specific mRNA levels in vaginal tissue.

**Results:**

The epigenome-wide scan suggested differences in methylation between soy formula–fed and cow formula–fed infants at three CpGs in the gene proline rich 5 like (*PRR5L*) (*p* < 10^4^). Pyrosequencing of the two feeding groups found that methylation levels progressively diverged with age, with pointwise differences becoming statistically significant after 126 days. Genistein-exposed mice showed a 50% decrease in vaginal *Prr5l* mRNA levels compared to controls.

**Conclusions:**

Girls fed soy formula have altered DNA methylation in vaginal cell DNA which may be associated with decreased expression of an estrogen-responsive gene.

**Citation:**

Harlid S, Adgent M, Jefferson WN, Panduri V, Umbach DM, Xu Z, Stallings VA, Williams CJ, Rogan WJ, Taylor JA. 2017. Soy formula and epigenetic modifications: analysis of vaginal epithelial cells from infant girls in the IFED study. Environ Health Perspect 125:447–452; http://dx.doi.org/10.1289/EHP428

## Introduction

Soy formula use during infancy has been associated with altered age at menarche (Adgent et al. 2012; D’Aloisio et al. 2013) and identified as a risk factor for uterine fibroids (D’Aloisio et al. 2012) and endometriosis (Upson et al. 2015). These associations might be attributed to exposure to the phytoestrogen genistein, which is present in high amounts in soy formula and binds to the estrogen receptor (ER) with highest affinity for ERβ (Kuiper et al. 1998). Neonatal rodents exposed to genistein show alterations in their estrous cycle, behavior, ovarian function, and reproductive tract (reviewed by Jefferson et al. 2012), as well as increased risk of tumors and possibly autoimmune diseases (Klein et al. 2002; Newbold et al. 2001). Short-term dietary exposure to genistein also alters DNA methylation patterns in mice (Day et al. 2002).

In the United States, about 12% of formula-fed infants are given soy formula (Rossen et al. 2016). Although soy formulas are considered to be safe, infants fed soy formula have blood concentrations of genistein that overlap with those showing biologic effects in rodents (Cao et al. 2009; McCarver et al. 2011), raising concern about possible latent or subclinical effects. As yet, few data are available for examining this hypothesis. A prospective observational study of soy formula, cow milk formula, and breastfed infants in the central United States found no difference in mental development status at 1 year of age between infants fed soy formula and those fed cow milk formula (Andres et al. 2012). In a follow-up study, reproductive organ size was compared between children who had been fed different formulas or breast milk; again, no statistically significant differences were found (Andres et al. 2015). However, reproductive organ size measurements may not be sensitive markers of long-term estrogen effects. A pilot study with a focus on the reproductive tract (Bernbaum et al. 2008) and preliminary data from the subsequent longitudinal study (Adgent et al. 2014) found that infant girls fed soy formula had vaginal cytological changes consistent with estrogen exposure. We hypothesized that early-life genistein exposure from soy formula might lead to later changes in the reproductive system through epigenetic alteration of reproductive tract tissues. Here we report on differences in DNA methylation patterns in vaginal epithelial cells between infant girls being exclusively fed either soy formula or cow milk formula during their first months of life.

## Methods

### Human Study Sample

All participants were part of the Infant Feeding and Early Development (IFED) Study, a longitudinal cohort study designed to identify differences in estrogen-responsive outcomes in infants fed cow milk–based formula, soy-based formula, or breast milk. Between August 2010 and March 2014, IFED enrolled mothers in the third trimester of pregnancy or within the first 72 hr postpartum and followed their infants prospectively. Feeding group was determined by the mother’s intention to feed a simple, exclusive regimen from birth; mother–infant pairs were excluded if their feeding method changed during follow-up. Eligible mothers had to speak English, be ≥ 18 years of age, and have no history of gestational diabetes, thyroid dysfunction, or other endocrine disorders. Eligible infants were healthy singletons, born between 37 and 42 weeks of gestation with a birth weight between 2,500 and 4,500 g. Mothers provided written informed consent for both themselves and their infants. The IFED study protocol was approved by the institutional review boards at the National Institute of Environmental Health Sciences and the Children’s Hospital of Philadelphia, the clinical site of the study.

All infant girls were followed until 9 months of age. Visits occurred every 2 weeks until 1 month of age and then every 4 weeks until end of follow-up. At each visit, infants had a physical examination and had biological samples collected, including a swab taken from the introitus of the vagina for cytological analysis. Methods related to swab collection and sample treatment have been previously described (Adgent et al. 2013). The study enrolled 410 mother–infant pairs, 397 of which contributed at least one swab sample. Two hundred eighty-three infant boys and girls completed the full course of the study (70 breast milk–fed, 111 cow formula–fed, and 102 soy formula–fed).

Our study used DNA extracted from cytology swabs collected after 17 December 2012. At this time, recruitment and follow-up of breastfed infants had concluded making those samples (all collected before 17 December 2012) unavailable for our analysis. The IFED study also enrolled boys who had swabs taken from the urethral meatus. DNA yields from the urethral samples were poor, so we excluded boys from the methylation study. Therefore, our analyses included only samples from cow milk– and soy formula–fed girls.

### Human Sample Processing

DNA was extracted from polyester swabs stored in SurePath Preservative (BD Diagnostics, Durham, NC) using the QIAamp DNA blood mini kit (Qiagen). After extraction, 237 of 464 available samples yielded sufficient DNA for further analysis. All extracted DNA was bisulfite converted using the EZ DNA methylation kit (Zymo Research) according to the manufacturer’s protocol. The 20 swabs yielding most DNA were used for epigenome-wide DNA methylation analysis with Infinium HumanMethylation450 BeadChip arrays (Illumina Inc.), including 2 samples run in duplicate. Input DNA ranged from 200 to 500 ng, which is less than the 500 ng recommended by the manufacturer. Additional details are provided in “DNA extraction and quantification” and “Epigenome wide array analysis” in the Supplemental Material.

The remaining 217 samples, from 52 girls, were used for pyrosequencing as described in “Pyrosequencing analysis” in the Supplemental Material and Table S1. During follow-up, 2 of the 52 infants changed feeding method and were therefore excluded, yielding a final sample size of 214 samples from 50 infant girls (155 from 28 soy formula–fed and 59 from 22 cow formula–fed). See Figure S1 for a complete description of sample selection. To provide direct comparison to 450K results we also pyrosequenced 10 samples that had been successfully analyzed on the 450K platform.

### Methylation and Gene Expression

The relationship between DNA methylation and gene mRNA expression was examined using publically available Cancer Genome Atlas (TCGA) correlation analyses deposited at Broad GDAC Firehose (https://gdac.broadinstitute.org/; no licensing approval required).

### Mouse Model

Animals were handled according to National Institutes of Health (NIH)/National Institute of Environmental Health Sciences (NIEHS) guidelines under approved animal care and use protocols. Timed pregnant CD-1 mice were obtained from the in-house breeding colony at NIEHS (Research Triangle Park, NC), housed in a temperature-controlled environment (21–22°C) under a 12 hr light:12 hr dark cycle, and fed NIH-31 diet. Sixty female pups were treated by subcutaneous injections (0.02 mL) on the day of birth (postnatal day 1) through postnatal day 5 with either genistein 50 mg/kg per day dissolved in corn oil (*n* = 30) or with corn oil alone (controls; *n* = 30) as described previously (Doerge et al. 2002). Treated mice were sacrificed on postnatal day 5 (20 exposed/20 control) and postnatal day 22 (10 exposed/10 control). Ten additional female pups were sacrificed on the day of birth without any prior treatment. Mouse vaginal tissue was collected and stored at –80°C. RNA extraction and expression analysis are described in “RNA extraction” and “Gene expression analysis” in the Supplemental Material.

### Statistical Analysis

Demographic characteristics were compared between feeding groups using chi-square and Fisher’s exact tests.

Before analyzing our EWAS (epigenome-wide association study) data we excluded 58,840 CpGs that mapped to multiple target regions, included SNPs, or were located on the X or Y chromosome. We then tested the association between feeding regimen (i.e., soy or cow milk) and DNA methylation (β-value) at each of the remaining 427,097 CpG sites from the genome-wide array using a robust linear regression model employed via the R package MASS (Venables and Ripley 2002) without correction for multiple comparisons.

To confirm our findings from the 450K analysis of human vaginal cell DNA we used pyrosequencing to measure percent DNA methylation at two specific CpG sites in the gene proline rich 5 like (*PRR5L*). We created a single summary methylation (M) value (denoted M^^—^^) for each sample by averaging four individual M values: those from duplicate runs for each of the two CpGs. We transformed M^^—^^ to logit(M^^—^^) = ln[M^^—^^/(1 – M^^—^^)] and used logit(M^^—^^) as the outcome in our statistical analysis. This transformation ensures that, upon back-transformation to the original scale, fitted M values will remain within the desired (0–1) range. We estimated age trajectories for logit(M^^—^^) for all subjects, and separately for each feeding group, using mixed-model techniques to account for variability both within and among subjects. We represented each mean trajectory as a natural cubic spline with three equally spaced knots using the square root of age (days at sample collection) as the predictor; knot locations were common to both feeding groups. We accounted for variability among subjects by declaring all spline coefficients except the intercept to be random with an unstructured covariance matrix, in effect allowing each subject her own trajectory. Our model allowed the two feeding groups to have different within-subject variances. Regarding the logit(M^^—^^) as having a normal distribution, we fit this mixed model using PROC GLIMMIX in SAS version 9.3 (SAS Institute Inc., Cary, NC). We calculated back-transformed fitted means, variances, and confidence limits using Taylor series methods.

Gene expression was evaluated in the mouse model. Using newborn mice as a reference we quantified relative levels of *Prr5l* mRNA in mouse vaginal tissue. We compared the mean mRNA levels of *Prr5l* between the following mouse groups: newborn versus postnatal day 5 exposed, newborn versus postnatal day 5 controls, and postnatal day 5 exposed versus postnatal day 5 controls with the Mann–Whitney *U* test.

## Results

### Epigenome-Wide Analysis

To identify differentially methylated loci in soy formula–fed versus cow formula–fed infant girls, we used the Illumina HumanMethylation450 BeadChip, which tags 485,577 CpG sites across the genome. Perhaps reflecting suboptimal input DNA, nine samples and one duplicate failed quality control metrics and were eliminated from subsequent analysis. Eleven vaginal-cell DNA samples and one duplicate provided high-quality methylation data for genomic analysis, corresponding to 4 soy formula–fed and 6 cow formula–fed subjects (1 cow formula–fed subject was sampled once at 20 weeks and in duplicate at 24 weeks of age). The demographic characteristics of the mothers of these 10 infants were similar to those described in Table 1, with 6 of 10 mothers identified as black, 7 of 10 mothers 21–30 years of age, and 7 of 10 with a high school education or less. The samples from 4 soy formula–fed girls were from younger ages (4–8 weeks of age) than the six samples from cow formula–fed girls (20–32 weeks of age).

**Table 1:** Participant characteristics.

Characteristic	Cow formula [*n* (%)]	Soy formula [*n* (%)]	*p*-Value^*a*^
Total	22 (100)	28 (100)
Maternal age (years)
20 or less	4 (18)	4 (14)	0.78
21–30	12 (55)	18 (64)
≥ 31	6 (27)	6 (21)
Child’s race
Black	15 (68)	20 (71)	0.74
White	5 (23)	4 (14)
Multiple/other	2 (9)	4 (14)
Mother’s race
Black	17 (77)	20 (74)	0.79
White	5 (23)	7 (26)
Missing (unknown)	0	1
Maternal education
High school/GED^*b*^ or less	15 (68)	14 (50)	0.19
Some college or more	7 (32)	14 (50)
^***a***^*p*-Values are from chi-square tests (maternal age, maternal education) or Fisher’s exact tests (mother’s race, child’s race). ^***b***^General Educational Development.			

**Figure 1:**
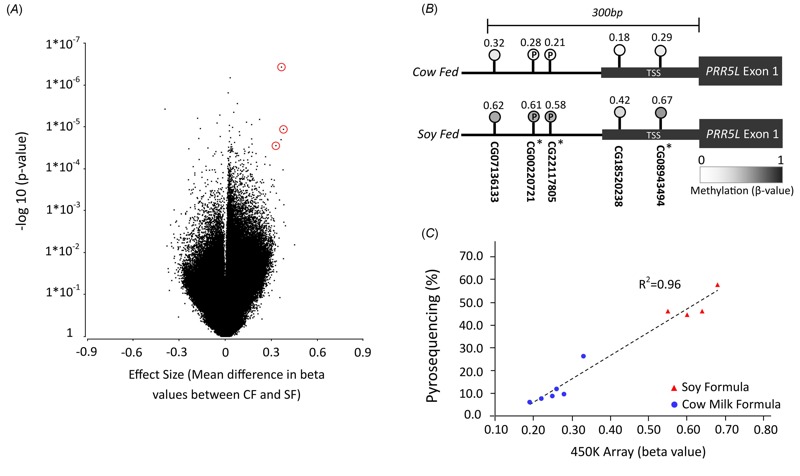
(*A*) Data from EWAS analysis. Volcano plot depicting all CpGs present on the 450K methylation array: log_10_ (*p*-value) plotted against effect size. Three CpGs in the gene* PRR5L* (circled) were identified as having small *p*-values in combination with large effect sizes. (*B*) Data from EWAS analysis. The 5’ region of *PRR5L* transcript variant 4 (Chr 11) showing CpGs and methylation levels obtained from 450K arrays and the three CpGs with largest effect sizes and smallest *p‑*values (*) from the volcano plot. CpGs validated by pyrosequencing are marked with P. DNA methylation levels were significantly higher in vaginal DNA samples from soy formula–fed girls. (*C*) Initial replication of 450K array results by pyrosequencing. All 11 individual samples that yielded usable data from the 450K array were re-run using pyrosequencing specific for cg00220721 and cg2211705. One sample (cow formula–fed) failed in the pyrosequencing run due to low input amount. The other 10 showed strong correlation (*R* ^2 ^= 0.96) between the average two CpGs from array versus pyrosequencing measurements.

Five CpG sites had *p*-values < 10^–4^ and also had effect sizes (difference in mean β-value between cow formula–fed and soy formula–fed) of > 0.3 (Figure 1A). Cg13935577 is located in the promoter region of *BTBD11* on chromosome 12 and cg20103692 is in *MAS1L* on chromosome 6. The three remaining sites (cg00220721, cg22117805, and cg08943494), including the site with the smallest *p*-value (cg22117805), are all located within 200 bp of one of the annotated transcriptional start sites of *PRR5L;* all three had β-values that were low in cow formula–fed girls (< 0.3) and high in soy formula–fed girls (> 0.5) (Figure 1B). Taken together, the small consistent *p*-values, large effect sizes, and close proximity of the three CpG sites to a *PRR5L* transcriptional start site led us to investigate these CpGs in a larger study that sought to assess both formula and age effects.

### Pyrosequencing

The two *PRR5L* CpGs with closest proximity to each other (12 bp apart) were selected for pyrosequencing analysis (cg00220721 and cg22117805). Methylation levels at these two sites were highly correlated (*R* ^2^ = 0.95) on 450K array analysis and the average methylation at the two sites on 450K analysis was in turn highly correlated with the average methylation obtained by pyrosequencing (*R* ^2^ = 0.96, Figure 1C). We did not assess any additional CpGs at this or other loci.

**Figure 2:**
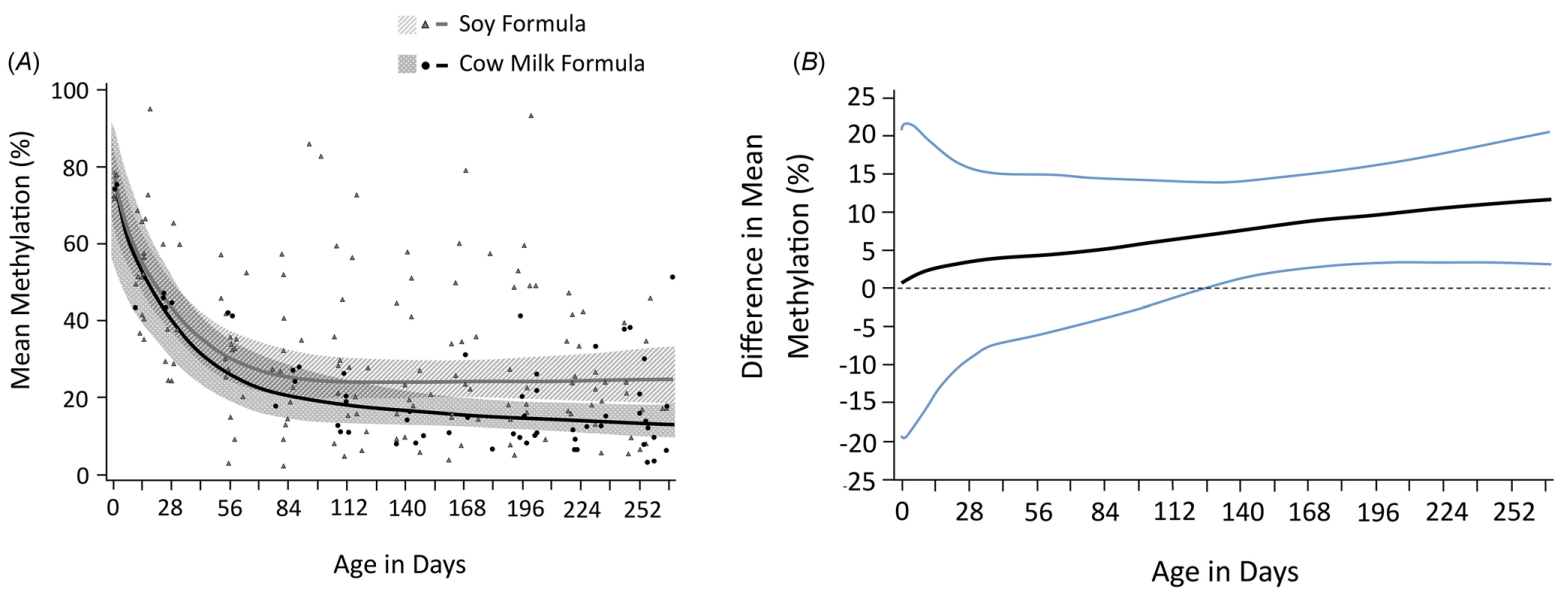
(*A*) Data from pyrosequencing. Replication of the top two *PRR5L* CpG sites by pyrosequencing. Each symbol represents the average of the two adjacent CpG methylation values from two separate pyrosequencing runs for each sample (triangles = soy formula–fed girls; solid dots = cow formula–fed girls). Lines represent the fitted mean trajectory for the corresponding feeding group; shading represents 95% pointwise confidence intervals. Estimated mean trajectories are natural cubic splines with three knots fitted to the data via mixed-model techniques. (*B*) Data from pyrosequencing. The difference between the fitted mean methylation at individual time points. The black line represents the difference between soy and cow formula values calculated from Figure 2A at each time point. The blue lines represent 95% pointwise confidence intervals for each time point.

To determine whether we could replicate results from the epigenome-wide analysis in independent samples and to investigate age effects, we used DNA from 214 vaginal-cell DNA samples (distinct from those included in the epigenome-wide analysis) from 50 infant girls (Table 1). Of the 214 samples examined with pyrosequencing, our final analysis included 205 samples (147 soy formula–fed and 58 cow formula–fed) from 49 girls, excluding 9 samples that failed in the duplicate sequencing runs for both sites. Mean methylation levels in the two feeding groups were similar and high, near 0.8, at the time of birth. Methylation levels decreased with increasing age (*p* < 0.001) but soy formula–fed girls maintained higher mean methylation levels compared with cow formula–fed girls (Figure 2A). Although the overall trajectories for soy formula– and cow formula–fed girls did not differ significantly (*p* = 0.25), methylation differences calculated at each time point (without correction for multiple comparisons) were statistically significant (*p* < 0.05) beginning about 126 days after birth (Figure 2B).

### *PRR5L* Gene Expression

To determine the relationship between methylation and mRNA expression of *PRR5L*, we examined publically available Cancer Genome Atlas (TCGA) data through the Broad GDAC Firehose database. DNA methylation at cg0022072 is significantly negatively correlated with *PRR5L* mRNA level with Spearman correlations of –0.53 to –0.55 (see Table S2) (Broad Institute TCGA Genome Data Analysis Center 2015a, 2015b).

**Figure 3:**
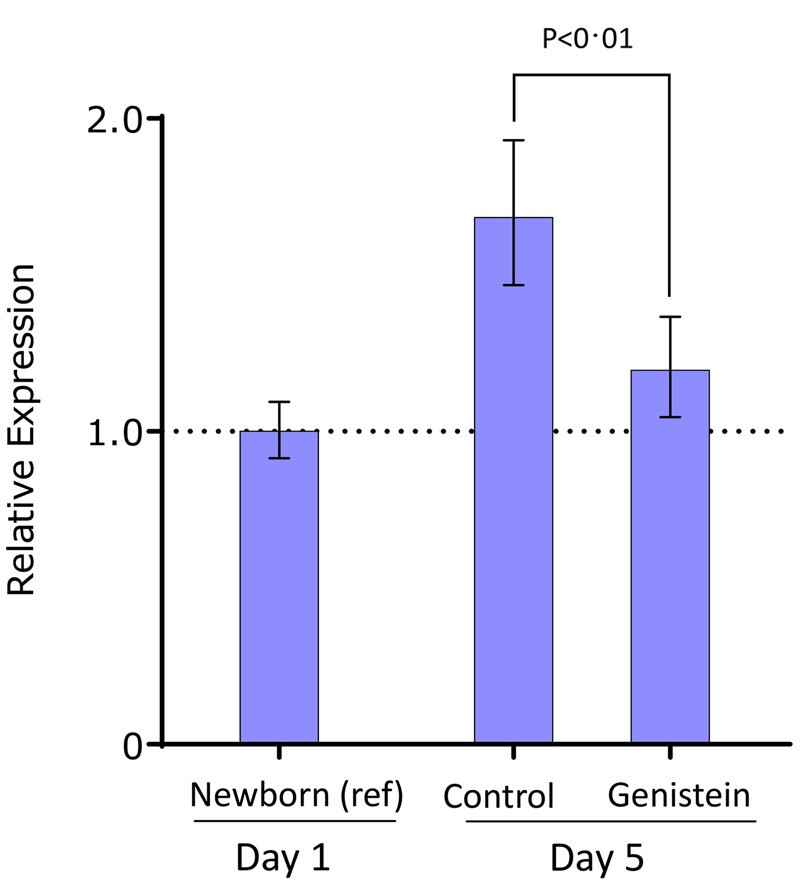
Relative expression of mouse *Prr5l*. Mean expression in untreated newborn mice was used as the reference (ref) level. Error bars represent standard error of the mean. Mice were treated with daily subcutaneous injections of genistein (50 mg/kg) or vehicle control on postnatal days 1–5.

To determine whether expression of *Prr5l* was affected by genistein exposure *in vivo*, we used a mouse model in which neonatal mice were exposed to genistein at doses that produced serum concentrations similar to those in human infants fed soy formula (Cao et al. 2009; Doerge et al. 2002). Relative to untreated newborn female mice, expression of *Prr5l* was higher in both exposed and control mice at postnatal day 5, but genistein-treated mice had significantly lower expression (*p* < 0.01) than control mice (Figure 3). By postnatal day 22, *Prr5l* expression had fallen more than 10-fold and did not differ by prior treatment (data not shown). The vaginal preparations from mouse that were used in this analysis include blood, connective tissue, and other cell types that may be less estrogen-responsive and therefore underestimate the effect of treatment on gene expression. These results from both human tumor tissue and mouse vaginal tissue suggest that the effect of genistein on DNA methylation in humans would be consistent with a corresponding decrease in expression of the *PRR5L* gene early in life.

## Discussion

Here we find that girls fed soy formula from birth exhibited higher DNA methylation at a specific gene locus in their vaginal tissue than did girls fed cow formula. Methylation differences were observed at CpG sites flanking a *PRR5L* transcriptional start site. These differences were confirmed by pyrosequencing of vaginal DNA samples that had been serially collected over 9 months following birth. Pyrosequencing revealed that DNA methylation at *PRR5L* was high at birth and fell rapidly in the 2 months after birth. This drop in methylation coincides with the infant’s rapidly falling exposure to maternal estrogens (Bidlingmaier et al. 1973). Compared with cow formula–fed girls, phytoestrogen-exposed soy-fed girls maintained higher methylation levels over time. Correlation between DNA methylation at cg0022072 and decreased gene expression was confirmed using deposited human data from the TCGA database. The long-term persistence of increased *PRR5L* DNA methylation in soy-fed infants and the health consequences, if any, remain unknown.

Relatively little is known about *PRR5L*, which encodes a component of the mTORC2 complex. The PRR5L protein (also known as protor-2) suppresses mTORC2-mediated activation of protein kinase C delta, which promotes fibroblast migration (Gan et al. 2012). PRR5L also promotes apoptosis via activation of the pro-inflammatory cytokine, tumor necrosis factor alpha (TNFα) (Thedieck et al. 2007). When PRR5L dissociates from the mTORC2 complex in response to mTORC2 activation, it binds to tristetraprolin (TTP), an RNA-binding protein that mediates sequence-specific degradation of mRNAs (Carrick et al. 2004; Holmes et al. 2012). PRR5L interaction with TTP appears to be required for proper TTP localization to cytoplasmic granules where mRNA processing occurs; in the absence of PRR5L, TTP does not degrade its target mRNAs. One of the best-known mRNA substrates for TTP is TNFα: Mice lacking TTP have high levels of TNFα due to low mRNA turnover, leading to severe arthritis and cachexia (Taylor et al. 1996). This link between PRR5L, TTP, and TNFα is interesting in light of recent studies demonstrating the association of a SNP (rs4755450) in *PRR5L* with juvenile idiopathic arthritis, and a microarray study showing that down-regulation of *PRR5L* was associated with osteoarthritis in adults (Chiaroni-Clarke et al. 2014; Wang et al. 2015). These findings suggest that suppression of *PRR5L* expression might promote TNFα-associated autoimmune diseases, including common conditions such as asthma, rheumatoid arthritis, psoriasis, or inflammatory bowel disease.

Using a murine model, we demonstrated that *Prr5l* is an estrogen-regulated gene, with genistein significantly suppressing *Prr5l* expression in vaginal tissue of mice exposed neonatally. This regulation is perhaps not surprising given results of chromatin immunoprecipitation sequencing showing that the mouse *Prr5l* gene locus has three distinct sites of ERα binding (Hewitt et al. 2012). Genistein affects mTOR signaling, leading to increased interest in its use in cancer prevention and treatment (reviewed by Ahmad et al. 2013), but epigenetic modification of *PRR5L* has not been previously reported.

Plant estrogens, along with other chemicals with estrogenic or other hormonal activity, are often classified as “endocrine disrupters.” Under the endocrine disruption hypothesis, exposure to certain cosmetics, plasticizers, pesticide residues, and dietary and other agents cause subtle alterations in endocrine function, leading to subsequent adverse health effects. Evidence exists for this hypothesis in whole animal model systems and in wildlife, with exposure during the critical perinatal period producing the strongest effects (Diamanti-Kandarakis et al. 2009). But even for the best-studied agents, those that exhibit estrogenicity, human epidemiologic data of health consequences have remained tenuous: for example, the inference that endocrine disruptors may be responsible for secular trends in sperm count, infertility, and obesity (Bergman et al. 2013). In part, the uncertainty in the human evidence reflects the substantial epidemiologic challenge of connecting very low-dose exposures during infancy and childhood to health effects that may only become manifest years later in adolescents and adults. Soy formula feeding of infants provides a useful window into this challenge because it involves early-life exposure to much higher levels of estrogenic compounds than would come from other putative endocrine disrupters (Behr et al. 2011; Riu et al. 2008).

Recent epidemiologic studies have shown that soy formula feeding is associated with alterations in reproductive tract structure and function, including occurrence of uterine fibroids (D’Aloisio et al. 2012), endometriosis (Upson et al. 2015), and early age at menarche (Adgent et al. 2012). In rodents, perinatal genistein exposure can cause a variety of subsequent adverse consequences to the reproductive system including altered estrous cycle, subfertility/infertility, delayed vaginal opening, ovarian dysfunction, and uterine adenocarcinoma (Jefferson et al. 2002; Newbold et al. 2001; Patisaul et al. 2014), along with systemic effects including development of obesity (Strakovsky et al. 2014). Many of these effects can be observed at serum concentrations similar to those experienced by infants fed soy formula (Cao et al. 2009). Other xenoestrogens can produce delayed health effects in humans, with the best-studied example being prenatal diethylstilbestrol (DES) exposure and vaginal cancer in adolescence (Hoover et al. 2011). Although the mechanism by which soy formula produces late reproductive effects in humans remains unknown, epigenetic changes have been proposed as a mechanism by which DES acts (Hilakivi-Clarke et al. 2013). Indeed, neonatal mouse exposure to DES induces epigenetic changes in the uterus that are persistently maintained and are associated with altered gene expression in adults (Jefferson et al. 2013). In addition, prenatal exposure to genistein leads to gene silencing via DNA methylation in the agouti mouse model (Dolinoy et al. 2006), along with permanent down-regulation of estrogen-responsive genes and hypermethylation of repetitive elements in prenatally exposed adult animals (Vanhees et al. 2011). Postnatal genistein exposure is associated with hypermethylation and decreased gene expression in relation to obesity in a murine model (Strakovsky et al. 2014). Epigenetic changes are also a prominent feature of cancer cells, and phytoestrogens are receiving increasing attention, including clinical trials, as epigenetic reprogrammers for cancer prevention and treatment in adults (Greenwald 2004; Pudenz et al. 2014). But what might be positive attributes in the setting of adult cancer may have different consequences in pre- and postnatal life where epigenetic landscapes are rapidly shifting (Gluckman et al. 2008). Phytoestrogens affect epigenetic programs in differentiating embryonic stem cells (Sato et al. 2011), developing embryos (Chan 2009; Dolinoy 2008), and, as we observed here, infants who consume soy formula.

According to the American Academy of Pediatrics, soy formula is specifically indicated for galactosemia and lactase deficiency (Bhatia et al. 2008). Families whose children do not have these conditions use it for a variety of reasons, such as successful use of soy formula products with a previous child, dietary practices, or family recommendations (Stang et al. 2010). In addition, soy formula is often a second formula given to children several months old after cessation of breastfeeding or in response to new onset of gastrointestinal symptoms. These children may receive lower doses or be less susceptible to epigenetic reprograming than the infant girls included in this study. Although the epigenetic data presented here will augment this discussion, we do not believe they constitute a clear contraindication for soy formula use.

Our study was limited to a single tissue from girls, it examined only a small proportion of the approximately 28 million CpG sites in the genome, and it did not examine non-CpG methylation or other epigenetic modifications. Consequently, the associations we find between soy formula and increased methylation at *PRR5L* should be viewed as an initial and exploratory example of such effects rather than a complete catalogue. Rapid advances in DNA and RNA sequencing technologies can provide more comprehensive coverage of the epigenome and effects on gene expression, but such assays were not possible in the present study because of the limited number of cells available for analysis.

In summary, we found epigenetic differences in serial samples of vaginal cells from infant girls fed soy formula compared with those fed cow formula. This finding is consistent with the ability of genistein, the principal phytoestrogen in soy formula, to act as an estrogen and to produce epigenetic alterations in animal models. The search for epigenetic effects could be extended to additional regions of the genome, other putative endocrine disruptors, different points in life following exposure, and different tissues. Our results provide additional support for the hypothesis that epigenetic modification may be a mechanism by which early-life exposures lead to later-life health effects.

## Supplemental Material

(490 KB) PDF
