# Supplementary material for: Soy Formula and Epigenetic Modifications: Analysis of Vaginal Epithelial Cells from Infant Girls in the IFED Study
Source: Environ Health Perspect. 2016 Aug 19;125(3):447–52. doi: 10.1289/EHP428 (PMC5332195; doi:10.1289/EHP428)
Supplement: (490 KB) PDF [file EHP428.s001.acco.pdf]

Environ Health Perspect
DOI: 10.1289/EHP428

Note to readers with disabilities: *EHP* strives to ensure that all journal content is accessible to all readers. However, some figures and Supplemental Material published in *EHP* articles may not conform to 508 standards due to the complexity of the information being presented. If you need assistance accessing journal content, please contact ehp508@niehs.nih.gov. Our staff will work with you to assess and meet your accessibility needs within 3 working days.

Supplemental Material

Soy Formula and Epigenetic Modifications: Analysis of Vaginal Epithelial Cells from Infant Girls in the IFED Study

Sophia Harlid, Margaret Adgent, Wendy N. Jefferson, Vijayalakshmi Panduri, David M. Umbach, Zongli Xu, Virginia A. Stallings, Carmen J. Williams, Walter J. Rogan, and Jack A. Taylor

Table of Contents: Page

DNA extraction and quantification 2

Epigenome wide array analysis 2

Pyrosequencing analysis 3

RNA extraction 4

Gene expression analysis 4

Table S1. Pyrosequencing primers 5

Table S2. Correlation between DNA methylation and gene expression 5

Figure S1. 6

References 7

DNA extraction and quantification

All DNA extractions were performed using the QIAamp DNA blood mini kit (Qiagen). Briefly: 10 µL RNAse A, 75 µL proteinase K solution and 600 µL Buffer AL were added to the samples which then were incubated 56˚C for a minimum of 2 hours. When the cell solution was clear the extraction proceeded according to the manufacturer's instructions. Extracted DNA was eluted in 50 µL buffer AE and quantified using the Qubit fluorometer (Life Technologies).

Epigenome wide array analysis

To assess the feasibility of using the urogenital DNA on the Illumina HumanMethylation450 BeadChip (Illumina Inc.) we performed a pilot study that included DNA extracted from 20 vaginal swabs (10 cow-formula-fed, 10 soy-formula-fed). All 20 samples were analysed for genome-wide DNA methylation patterns by the NIEHS Molecular Genomics Core. Equal numbers of samples from cow and soy formula fed infants were placed on each chip with random assignment of chip position. Each chip also contained one duplicate sample (with original on the opposite chip) and one methylation control (100% or 0%). DNA was hybridized to the array following the manufacturer's protocol and then scanned with an Illumina iScan (Illumina Inc.). Samples were excluded if either 1) average intensity value across Illumina's bisulfite internal control probes was < 3000 or 2) fewer than 95% of CpG probes were detectable (Illumina detection p-value <0.05). Raw intensity data were extracted using Illumina GenomeStudio software (version 2011.1). At each CpG site on the array, methylation status was determined as previously described (Harlid et al. 2014).

Pyrosequencing analysis

A Pyrosequencing assay for *PRR5L* was designed using Pyromark Assay Design (Qiagen) (Supplementary Table S1). DNA from 304 samples was then subjected to pyrosequencing analysis as follows: reaction mixtures (25 µL) containing bisulfite converted DNA, 5 pmol of each primer (forward and reverse) PCR buffer (Life Technologies), 3 mM MgCl2, 1 mM dNTP, and 0.8 units of Taq polymerase (Life Technologies) were heated to 95°C for 15 minutes, followed by 45 PCR cycles (95°C for 20 seconds, 58°C for 20 seconds and 72°C for 20 seconds) with a final extension at 72˚C for 5 minutes. Following PCR, the biotin labeled PCR product was hybridized to streptavidin-coated sepharose beads (GE Healthcare), and denatured in 0.2 M NaOH to provide a single-stranded sequencing template. Pyrosequencing primers (0.3 µmol/L) were annealed to the single-stranded template and the pyrosequencing was carried out using PyroMark Q96 MD System (Qiagen) according to the manufacturer's instructions. Percentage methylation was quantified using the Pyro Q-CpG Software (Qiagen). Multiple laboratory replicates of MCF7 cell line and commercially obtained DNA were run with each sequencing plate, providing intra-run CVs of 4.5% and 4.8% and inter-run CVs of 4.5% and 1.4% for CpGs 1 and 2 respectively. All samples were sequenced at both CpGs in independent runs on two separate days. Sample-specific average methylation level of the two CpGs had a correlation of 0.75 between run 1 and 2, and the within-sample correlation of the two CpGs has high for each run (correlations of 0.87 and 0.91 respectively). For each sample we averaged the results of the two CpGs and of the separate runs, excluding from the final analysis nine samples that failed in one of the runs.

RNA extraction

RNA from mouse vaginal tissue was extracted using the RNeasy mini kit (Qiagen) as follows: frozen tissue samples were transferred to 50 mL Falcon tubes containing 400 µL of buffer RLT plus. The tissue was immediately homogenized using a rotor-stator homogenizer for 30 seconds. The homogenized lysate was transferred to a DNAeasy spin column and centrifuged at full speed for 30 seconds. The flow through was transferred to a 2 mL microcentrifuge tube and used for RNA extraction according to the manufacturer's instructions. RNA was eluted in 100 µL of RNAse free water and RNA concentration was determined using a Qubit fluorometer (Life Technologies).

Gene expression analysis

cDNA was generated using SuperScript® first-strand synthesis kit (Life Technologies) according to the manufacturer's instructions. After reverse transcription, single stranded DNA concentrations were measured using the Qubit flourometer (Life Technologies). Fifteen ng of cDNA was used for PCR amplification of the target site that was performed in triplicate using pre-designed TaqMan gene expression assays for *Prr5l* (Mm01327547_m1) and *18S* (Mm03928990_g1). Runs were carried out on the ViiA™ 7 Real-Time PCR System (Life Technologies) according to the manufacturer's instructions. All measurements were normalized to *18S* and expression levels were calculated using the ΔΔCT method (Pfaffl 2001) for relative quantification.

Table S1. Pyrosequencing primers

Primers for *PRR5L* cg00220721 and cg22117805	Sequence
Forward Primer	5’- AATGGGAATTTGTTAGGGAATAGATG-3’
Reverse Primer	5’-Biotin- AACTAAAACTTTCCAACCTAACTACC-3’
Sequencing Primer	5’- AGGGAATAGATGTTATTGT-3’

Table S2. Correlation between DNA methylation and gene expression at cg00220721

Accessed from Broad Institute TCGA Genome Data Analysis Center. 2015

TCGA Tissue type	TCGA Cohort	Probe	Corr_Coeff	Pval	Qval	Expr_Mean	Meth_Mean
Thyroid Adenocarcinoma primary solid tumor	THCA	cg00220721	-0.53	<0.001	<0.001	6.33	0.76
Cholangiocarcinoma primary solid tumor	CHOL	cg00220721	-0.55	<0.001	<0.001	6.45	0.68

Figure S1

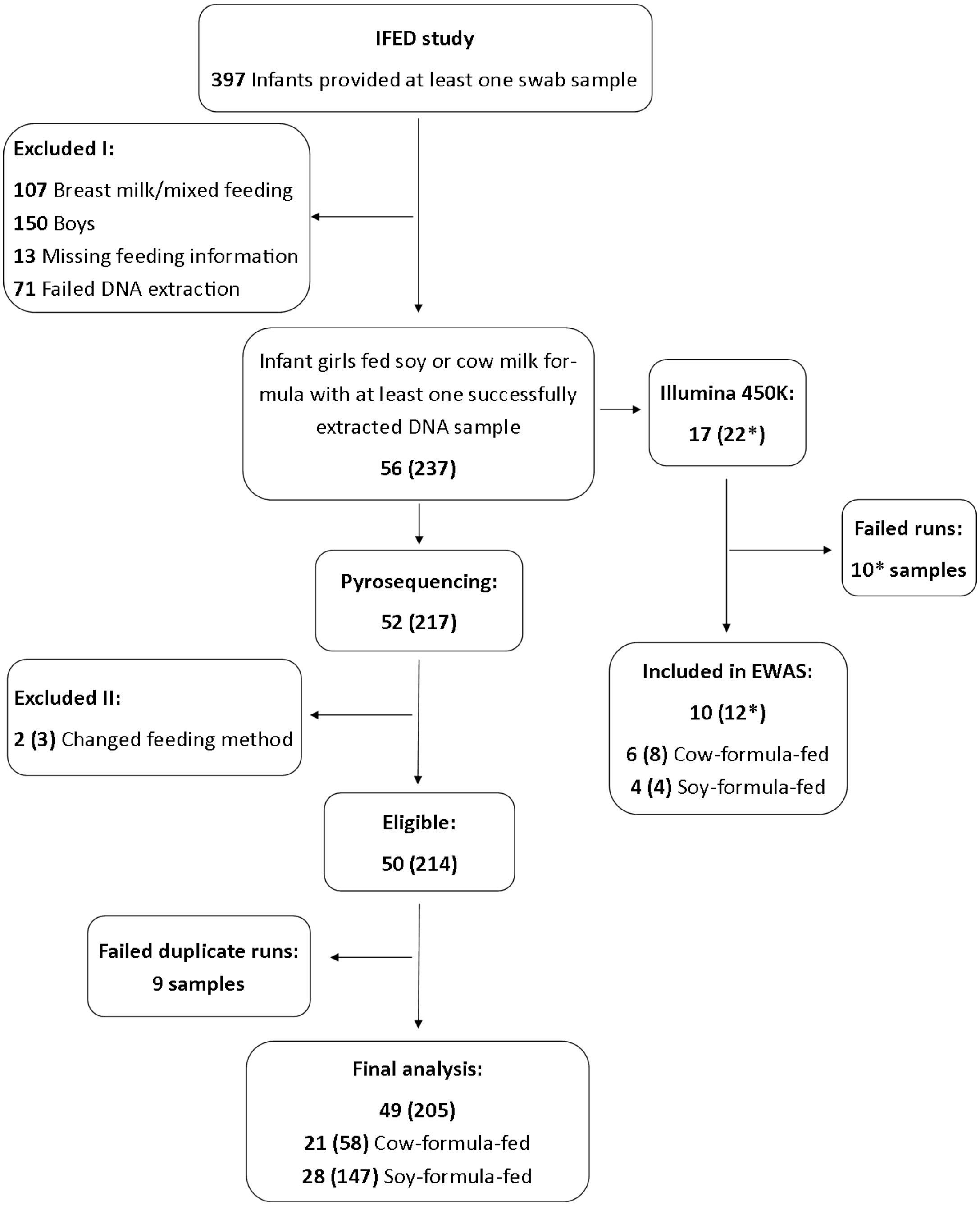

Figure S1: Flowchart describing selection and exclusion of samples from the original IFED study. Infants who provided at least one swab sample were eligible even if they did not complete the whole study. When suitable, number of samples is given in parenthesis. Samples "Included in EWAS" were not included in pyrosequencing "Final Analysis".

*Includes duplicate samples

References

Broad Institute TCGA Genome Data Analysis Center. 2015. Correlation between mRNA expression and DNA methylation. Broad Institute of MIT and Harvard. doi:10.7908/C1RV0MWM, 10.7908/C1WM1CQP [accessed 18 March 2016].

Harlid S, Xu Z, Panduri V, Sandler DP, Taylor JA. 2014. CpG sites associated with cigarette smoking: analysis of epigenome-wide data from the sister study. Environ Health Perspect 122: 673-8.

Pfaffl MW. 2001 A new mathematical model for relative quantification in real-time RT-PCR. Nucleic Acids Res 29:e45.
